# 43.2 MUSCARINIC M1 RECEPTORS: INVOLVEMENT IN THE PATHOPHYSIOLOGY AND TREATMENT OF SCHIZOPHRENIA

**DOI:** 10.1093/schbul/sby014.180

**Published:** 2018-04-01

**Authors:** Brian Dean, Shaun Hopper, Elizabeth Scarr

**Affiliations:** 1 Florey Institute for Neuroscience and Mental Health; 2 University of Melbourne

## Abstract

**Background:**

Evidence from postmortem CNS studies and a neuroimaging study suggest that, compared to controls, there are low levels of muscarinic receptors in a number of CNS regions from subjects with schizophrenia. Current data suggests the muscarinic M1 receptor is lower in the cortex of subjects with schizophrenia but other muscarinic receptors may be decreased in sub-cortical regions such as the striatum and hippocampus. In addition, it has been reported that ~25% of subjects with schizophrenia can be divided into a distinct sub-group because they have a marked decrease in cortical muscarinic M1 receptors (muscarinic receptor deficit schizophrenia (MRDS)). These findings have become of clinical significance because proof of principal data shows that treating subjects with schizophrenia with drugs that activate the muscarinic M1 receptor is effective in lessening the symptoms associated with the disorder.

**Methods:**

Published and unpublished data will be reviewed to challenge the hypothesis that drugs that activate the muscarinic M1 receptor will be useful in treating schizophrenia.

**Results:**

A proof of clinical trial has shown that treating subjects with treatment resistant schizophrenia with the muscarinic M1 and 4 receptor agonist, xanomeline, improves positive and negative symptoms as well as cognitive deficits. Moreover, it has more recently been reported that giving xanomeline on a transdermal patch with a peripheral muscarinic receptor antagonist can lessen the unwanted side effects of the drug to that of placebo. Relevant to these data is the finding that there is a sub-group of subjects with MRDS as the absence of cortical muscarinic M1 receptors in these receptors may make these subjects resistant to treatment with muscarinic M1 receptor agonists. However, novel studies using postmortem CNS from subjects with MRDS and non-MRDS has shown that whilst subjects with MRDS will likely be resistant to muscarinic M1 receptor orthosteric agonists (oxotremorine-M) they will, at least partially, respond to muscarinic M1 receptor allosteric agonists (AC-42) or positive allosteric modulators (BQCA).

**Discussion:**

Muscarinic receptor agonism appears to be a promising new treatment for schizophrenia. However, some subjects with MRDS may only respond to activation of the allosteric site on the muscarinic M1 receptor. Evidence from a neuroimaging study suggests subjects with MRDS can be identified whilst living. Hence, establishing the muscarinic receptor status of subjects involved in trials of muscarinic M1 receptor agonists may help in explaining varying levels of treatment responsiveness in subjects with schizophrenia. These conclusions, being directed by data from studies using postmortem CNS, reflect the need for drug discovery and delivery to be based on a growing understanding of the pathophysiology(ies) of schizophrenia.

